# Inhibitory Effect of Thai Purple Rice Husk Extract on Chemically Induced Carcinogenesis in Rats

**DOI:** 10.3390/molecules26020360

**Published:** 2021-01-12

**Authors:** Charatda Punvittayagul, Arpamas Chariyakornkul, Paweena Sankam, Rawiwan Wongpoomchai

**Affiliations:** 1 Department of Biochemistry, Faculty of Medicine, Chiang Mai University, Chiang Mai 50200, Thailand; Charatda.pun@cmu.ac.th (C.P.); arpamas.c@gmail.com (A.C.); 2 Research Affairs, Faculty of Veterinary Medicine, Chiang Mai University, Chiang Mai 50100, Thailand; 3 Sankamphaeng School, Saimun Sankamphaeng, San Kamphaeng, Chiang Mai 50130, Thailand; paweena.sankam@gmail.com

**Keywords:** 1,2-dimethylhydrazine, aberrant crypt foci, diethylnitrosamine, GST-P-positive foci, purple rice husk

## Abstract

This study investigated the cancer chemopreventive effects of an acidic methanol extract of purple rice husk on chemically induced carcinogenesis in rats. This purple rice husk extract (PRHE) had high polyphenol contents. Vanillic acid was a major phenolic compound in PRHE. Three major anthocyanins found in PRHE were malvidin-3-glucoside, peonidin-3-glucoside and cyanidin-3-glucoside. PRHE was not toxic and clastogenic in rats. The LD_50_ of PRHE was greater than 2000 mg kg^−1^ body weight (BW). The oral administration of 300 or 1000 mg kg^−1^ BW of PRHE for 28 days significantly decreased the number of micronucleated hepatocytes in diethylnitrosamine-initiated rats. The inhibitory mechanisms were associated with the reduction of cytochrome P450 2E1 expression and induction of some detoxifying enzymes in the liver. In addition, treatment with 500 mg kg^−1^ BW of PRHE for eight weeks did not induce preneoplastic lesions in the liver and colon. It significantly inhibited hepatic glutathione-*S*-transferase positive foci formation induced by diethylnitrosamine and 1,2-dimethylhydrazine by suppression of hepatocyte proliferation and induction of apoptosis. In conclusion, PRHE did not present toxicity, clastogenicity or carcinogenicity in rats. It exhibited cancer chemopreventive properties against chemically induced early stages rat hepatocarcinogenesis. Anthocyanins and vanillic acid might be candidate anticarcinogenic compounds in purple rice husk.

## 1. Introduction

Recently, gastrointestinal cancer incidence in Asia has been annually increasing because of dietary patterns and lifestyle changes, obesity and ageing [1]. Furthermore, cancer-control programs and early detection have been found useful in some Asian countries. Therefore, the prevention of cancer might be one of targeting strategies to reduce the incidence and mortality rates of cancer.

Numerous reports have shown evidence that bioactive compounds rich in natural products exhibit various biological activities to rejuvenate health and alleviate diseases [2]. Recently, the inedible parts of plants, such as the peel and husk, which contain high polyphenolic compound content, have been used for food supplements [3]. Punicalin from pomegranate husk presented free radical scavenging activity [4], while quercetin and protocatechuic acid methyl ester from Chinese hickory husk exhibited antioxidant activity and neuroprotective effects on SH-SY5Y cells [5]. In addition, ripe pistachio hull showed strong antioxidant capacities in in vitro models and cytoprotective properties on lymphocytes treated with *tert*-butyl hydroperoxide [6]. Polysaccharides from *Citrus sphaerocarpa* peels inhibited angiogenesis and cell migration in MDA-MB-231 breast cancer cells [7].

Rice (*Oryza sativa* L.) husk is a valuable by-product obtained from the rice milling process. It has been widely used as a fuel for biomass gasification and electricity generation and also as an animal feed [8]. Recent research has indicated that *p*-coumaric acid, ferulic acid, vanillic acid and caffeic acid have been identified as major phenolic acids in rice husk [9,10]. There are many anthocyanins which have been identified in pigmented rice husk, such as cyanidin-3-glucoside, peonidin-3-glucoside and malvidin-3-glucoside [11]. In addition, pigmented rice husk, which contains higher polyphenolic compounds than white rice, showed high free radical scavenging and antimutagenic activities [12,13]. Moreover, colored rice husk extract exhibited higher antioxidant and chemopreventive activities than white rice husk in MCF-7 breast cancer cell lines [14]. Momilactone B from rice husk presented cytotoxic and antitumor activities against human colon cancer cells [15]. Furthermore, lipophilic and hydrophilic extracts of purple rice husk presented antimutagenicity in a bacterial mutation assay and anticlastogenicity in a rat liver micronucleus test [10,11,16]. However, information on the anticarcinogenic potential of rice husk in animal models is unclear. Therefore, to investigate the inhibitory effect of purple rice husk on chemically-induced carcinogenesis in the liver and colon, we studied a liver micronucleus assay and a medium-term dual-organ carcinogenicity model in rats.

## 2. Results

### 2.1. Chemical Constituents in Purple Rice Husk Extract

**Figure 1:**
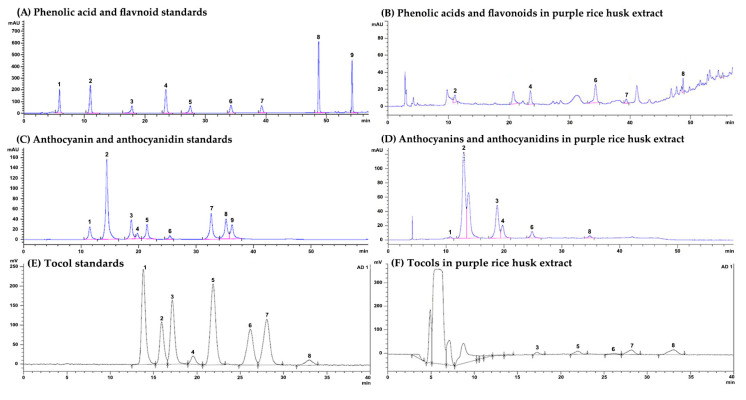
The HPLC chromatograms of (**A**) a mixture of phenolic acid and flavonoid standards: peaks 1, gallic acid; 2, protocatechuic acid; 3, catechin; 4, vanillic acid; 5, epicatechin; 6, *p*-coumaric acid; 7, ferulic acid; 8, rutin; and 9, quercetin, (**B**) phenolic acids and flavonoids in purple rice husk extract, (**C**) anthocyanin and anthocyanidin standards. Peaks 1, delphinidin-3-glucoside; 2, cyanidin-3-*O*-glucoside; 3, peonidin-3-*O*-glucoside; 4, malvidin-3-glucoside; 5, delphinidin; 6, cyanidin; 7, pelargonidin; 8, peonidin; and 9, malvidin, (**D**) anthocyanins and anthocyanidins in purple rice husk extract, (**E**) tocol standards. Peaks 1, δ-tocotrienol; 2, β-tocotrienol; 3, γ-tocotrienol; 4, α-tocotrienol; 5, δ-tocopherol; 6, β-tocopherol; 7, γ-tocopherol; and 8, α-tocopherol, (**F**) tocols in purple rice husk extract.

The contents of total phenolic compounds and total flavonoids in purple rice husk extract (PRHE) were 58.2 ± 0.49 and 28.4 ± 0.29 mg g^−1^ extract, respectively. The particular hydrophilic and lipophilic compounds contained in the extract are shown in Table 1. The chromatograms are shown in Figure 1. Small amounts of various forms of vitamin E were detected. α-Tocopherol was a major vitamin E found in PRHE. The most abundant phenolic acids contained in the extract were vanillic acid, *p*-coumaric acid and protocatechuic acid, while gallic acid, catechin, epicatechin and quercetin were not found. The major anthocyanins contained in the extract were malvidin-3-glucoside, peonidin-3-glucoside and cyanidin-3-glucoside, as well as free cyanidin. In addition, small amounts of delphinidin-3-glucoside and peonidin were also found in the extract, while pelargonidin, delphinidin and malvidin were not detected.

### 2.2. Acute Toxicity, Clastogenicity, and Anticlastogenicity of Purple Rice Husk Extract

No mortality or toxicity related clinical signs were observed in rats after a single oral dose of PRHE at a concentration of 2000 mg kg^−1^ body weight (BW). Food and water intake, organs, and body weight did not show any significant changes compared to the control group (data not shown). These results showed that PRHE had no toxicity to rats. The LD_50_ of the PRHE was greater than 2000 mg kg^−1^ BW.

**Figure 2:**
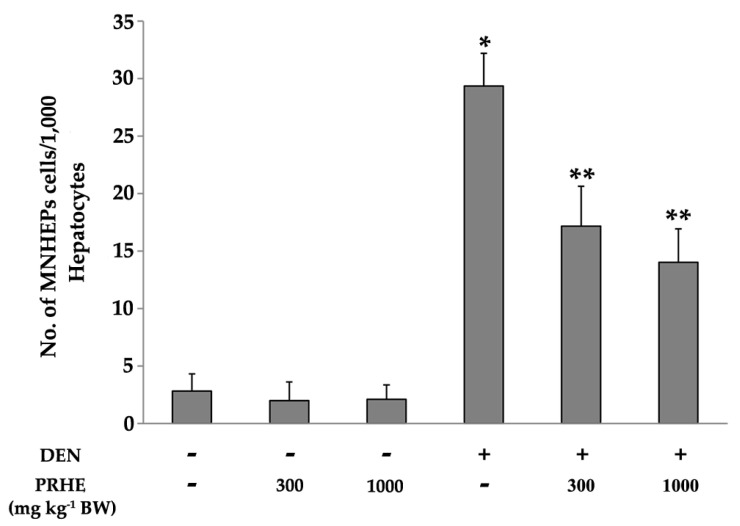
Clastogenicity and anticlastogenicity of PRHE in the liver of DEN-initiated rats. Value are expressed as mean ± SD. DEN: diethylnitrosamine 30 mg kg^−1^ body weight (BW), i.p. MNHEPs: micronucleated hepatocytes. PRHE: purple rice husk extract. * Significantly different from normal saline solution (NSS) group, *p* < 0.05. ** Significantly different from DEN group, *p* < 0.05.

The extract at concentrations of 300 or 1000 mg kg^−1^ BW had no effect on the mitotic index (data not shown) and did not induce micronucleus formation in rat liver. These findings showed that the extract had no clastogenicity in rat liver. Interestingly, treatment of 300 or 1000 mg kg^−1^ BW of the extract significantly reduced the number of micronucleated hepatocytes in diethylnitrosamine (DEN)-initiated rats (Figure 2). These findings demonstrated that PRHE presented anticlastogenicity against DEN-induced liver micronucleus formation in rats.

**Figure 3:**
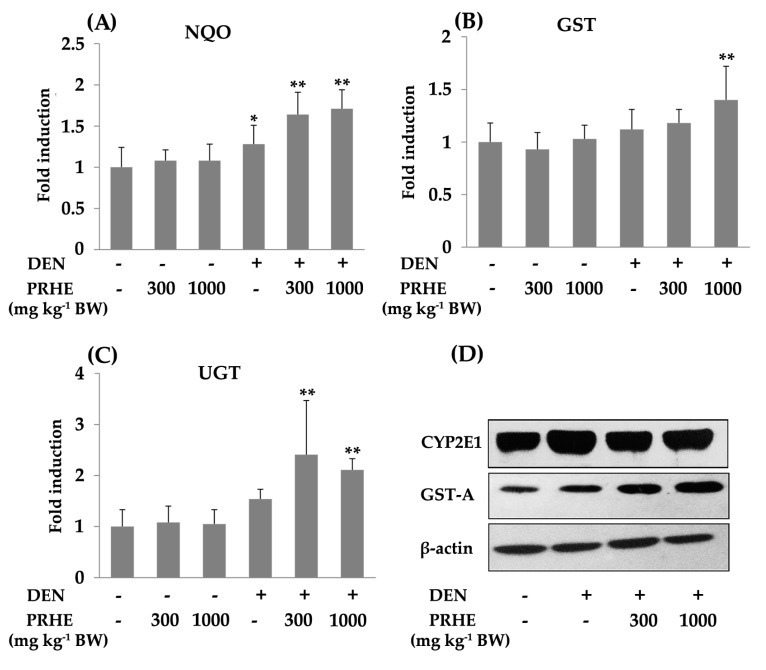
Effect of PRHE on the activity and expression of some hepatic xenobiotic-metabolizing enzymes in rats. The activity of (**A**) NQO: NADPH-quinone reductase, (**B**) GST: glutathione *S*-transferase, (**C**) UGT: UDP-glucuronyltransferase, (**D**) Western blotting of CYP2E1: cytochrome P450 2E1, GST-A: GST-alpha form. Values are expressed as mean ± SD. DEN: diethylnitrosamine 30 mg kg^−1^ BW, i.p. PRHE: purple rice husk extract. * Significantly different from negative control group, *p* < 0.05. ** Significantly different from positive control group, *p* < 0.05.

Furthermore, the oral administration of the extract at concentrations of 300 or 1000 mg kg^−1^ BW did not alter the activity of NADPH-quinone reductase (NQO), glutathione-*S*-transferase (GST) and UDP-glucuronyltransferase (UGT). Interestingly, at these concentrations, the extract significantly increased the activity of NQO, GST and UGT in DEN-initiated rats (Figure 3A–C). However, treatment with the extract did not affect the activities of CPR and heme oxygenase (HO) (data not shown). In addition, the extract significantly reduced the expression of CYP2E1 and increased the expression of GST-A enzymes when compared with the DEN-treated group (Figure 3D). These results suggest that PRHE presents cancer chemopreventive activity due to the reduction of phase I enzymes and induction of phase II enzymes in the DEN-induced initiation stage of rat hepatocarcinogenesis.

### 2.3. Carcinogenicity and Anticarcinogenicity of Purple Rice Husk Extract

PRHE at a dose of 500 mg kg^−1^ BW was given to investigate the anticarcinogenic potential on DEN- and 1,2-dimethylhydrazine (DMH)-induced preneoplastic lesion formation in the liver and colon of rats according to its anticlastogenic dosage between 300 and 1000 mg kg^−1^ BW of the extract. The general observations including body weight, water and food intakes and relative liver weight were not significantly different between treated groups and the negative control group (data not shown). These results indicate that PRHE at a dose of 500 mg kg^−1^ BW was not toxic to rats. PRHE did not induce the formation of hepatic GST-P-positive foci and colonic aberrant crypt foci (ACF), indicating no carcinogenicity in rat liver and colon. Interestingly, treatment with the extract after carcinogen treatment significantly decreased the number of GST-P-positive foci in rat liver but did not modulate the number of ACF in the colon. The results are summarized in Table 2.

Rats treated with DEN and DMH had significantly increased numbers of PCNA-positive hepatocytes in GST-P-positive foci and this did not influence apoptosis of the liver. Administration of PRHE reduced the number of PCNA-positive cells in both the preneoplastic lesion and surrounding areas when compared with the carcinogen induced group. In addition, the number of apoptotic cells in the liver of purple rice husk treated rats was higher than that in carcinogen-initiated rats (Table 3). Taken together, these results suggest that PRHE might suppress the early stages of rat hepatocarcinogenesis by reduction of cell proliferation and induction of cell apoptosis.

## 3. Discussion

Rice husk, an agricultural by-product obtained from the rice harvesting process, has been found several biological activities; however, the investigation on its anticarcinogenic effect is limited. The present study found that PRHE contained high amounts of phenolic acids, such as vanillic acid, *p*-coumaric acid and protocatechuic acid, which typically scavenge free radicals and play a major role in cancer prevention [17,18]. Anthocyanins, including malvidin-3-glucoside, peonidin-3-glucoside and cyanidin-3-glucoside, have been found to exhibit antioxidant and anticancer activities [19,20,21]. It might be suggested that the PRHE exerts anticarcinogenicity.

Several mechanisms of cancer chemopreventive agents acting on the initiation stage of carcinogenesis are involved in the modulation of phase I xenobiotic-metabolizing enzymes, induction of phase II xenobiotic-metabolizing and antioxidant systems [22]. Diethylnitrosamine, a hepatocarcinogen, is metabolized by cytochrome P450 2E1 (CYP2E1), to produce electrophilic metabolites, which further attack DNA, resulting in genotoxicity [23]. However, the reactive metabolites can be excreted from the body by conjugation with phase II enzymes such as NQO, UGT and GST [24]. PRHE presented anticlastogenic effects against DEN-induced hepatic micronucleus formation in rats. It inhibited CYP2E1 expression and induced the activities of UGT, GST and NQO. These results suggest that the anticlastogenic effect of purple rice extract on DEN-induced hepatic micronucleus formation might be caused by inhibition of phase I enzymes and induction of phase II enzymes in DEN metabolism.

An imbalance between cell proliferation and apoptosis is an important scenario for cancer development [25]. We found that the administration of PRHE during the promotion phase of DEN and DMH manipulation also reduced the number of GST-P-positive foci in the liver. Furthermore, PRHE remarkably decreased the number of PCNA-positive cells, a marker of cell proliferation and induced the number of apoptotic cells in the liver of DEN- and DMH-treated rats. These results might support evidence that the inhibition of cell proliferation and induction of cell apoptosis are necessary for cancer suppression [22]. Although the anticarcinogenic effect of PRHE was observed in the liver, this effect was not seen in the colon using this animal model. In general, phytochemicals undergo extensive biotransformation in the liver and are further conjugated with glucuronic acid, glutathione or sulfate before they are excreted from the body [26]. The colon is the second part of the digestive tract that absorbs polyphenols. The unabsorbable polyphenols from the small intestine will reach the colon, to be reabsorbed [27]. Based on these observations, some phytochemicals contained in the PRHE might be absorbed and be transported to the liver, where they act as anticarcinogens. At this dosage, their conjugated forms might not react with the neoplastic process in the colon resulting in non-anticarcinogenicity in the colon.

It is well-known that methanol is a good solvent for polyphenol extraction, especially in acidic conditions for anthocyanin-rich extract preparations [28]. In the present study, the acidified methanol extract of purple rice husk provided higher amounts of anthocyanins and anthocyanidins than those of non-acidified PRHE [11]. A previous report has indicated that cyanidin-3-glucoside and peonidin-3-glucoside presented antitumor activity in Lewis lung carcinoma cells in vivo [19]. In addition, anthocyanins extracted from Chinese blueberry presented anticancer activity in HepG2 by inhibiting cell proliferation and increasing cell apoptosis [29]. Furthermore, protocatechuic acid, a first-pass metabolite of cyanidin-3-glucoside, has presented in vitro anticarcinogenicity and hepatic GST induction in rats [30,31]. It is possible that anthocyanins containing in PRHE might be metabolized to protocatechuic acid and then induced the activity of GST in rat liver. These findings have been supported by our study that anthocyanins might be one of the anticarcinogenic agents in PRHE. Additionally, our previous study has indicated that vanillic acid, the highest phenolic acid in PRHE, exhibited anticlastogenicity against aflatoxin B_1_–induced micronucleus formation in rat liver by modulation of GST activity [10]. Moreover, vanillic acid has suppressed human colon cancer HCT116 cells via HIF-1α inhibition [32]. It could therefore be assumed that vanillic acid is one of the anticarcinogenic agents in the PRHE. Various vitamin E forms were also found in this extract but in low amounts. Although α-tocopherol was the largest amount in this extract, the previous investigation has demonstrated that it had no effect on both liver and colon carcinogenesis [33]. This shows that vitamin E might not be the active compound in purple rice husk. Based on these findings, anthocyanins and vanillic acid might be anticarcinogenic compounds in PRHE. However, forthcoming research should be targeted on these active compounds for a much deeper understanding.

## 4. Materials and Methods

### 4.1. Chemicals

Diethylnitrosamine and 3,3′-diaminobenzidine tetrahydrochloride hydrate were purchased from Sigma Aldrich (St. Louis, MO, USA). Collagenase type IV and 4′,6-diamidino-2-phenylindole (DAPI) were obtained from Invitrogen (Waltham, MA, USA). The 1,2-dimethylhydrazine dihydrochloride was purchased from TCI (Tokyo, Japan). The ApopTag Peroxidase in situ kit and methylene blue were obtained from Merck (Darmstadt, Germany). The Vectastrain ABC kit was obtained from Vector Laboratories, Inc. (Burlingame, CA, USA). The EnVision Doublestain system was provided by Dako (Glostrup, Denmark). All other chemicals were analytical grade.

### 4.2. Preparation of Purple Rice Husk Extract

The husk of purple rice (*Oryza sativa* var. *Indica*) cv. Kum Doisaket was obtained during the rice milling process. The rice was cultivated from August to November at the Lanna Rice Research Center, Chiang Mai University, Chiang Mai, Thailand. Briefly, purple rice husk was extracted twice with 0.1% HCl in methanol for 48 h. After filtration, the filtrate was evaporated under reduced pressure and lyophilized to obtain the acidic methanol extract of purple rice husk. The crude extract was used in the experiments.

### 4.3. Determination of Some Chemical Constituents in Purple Rice Husk Extract

The contents of total phenolic acids and total flavonoids in PRHE were determined by using colorimetric methods. To measure the total phenolic content, the extract was mixed with 50% Folin–Ciocalteu reagent and incubated at room temperature for 10 min. Then, 7% Na_2_CO_3_ was added, and the mixture was incubated at 45 °C for 15 min. The absorbance was measured at 765 nm. Gallic acid was used as a standard, and the content was expressed as mg of gallic acid equivalent per gram extract (mg GAEg^−1^ extract). The total flavonoid content was examined by the aluminum chloride colorimetric method, using catechin as a standard [34]. Briefly, the extract was mixed with the reaction mixture containing 5% NaNO_2_ and 10% AlCl_3_⋅6H_2_O and incubated at room temperature for 20 min. After that, 1M NaOH was added, and the absorbance was measured at 532 nm. The result was expressed as mg of catechin equivalent per gram extract (mg CEg^−1^ extract).

Tocols, phenolic acids, flavonoids, anthocyanins and anthocyanidins contents were also determined by high-performance liquid chromatography according to Punvittayagul et al., 2014 [11]. The tocols (α, β, γ and δ forms) were examined by using a Develosil C30 UG column (250 × 4.6 mm, 5µm) and eluted with methanol:distilled water (90:10). Each form of tocols was detected by a fluorescence detector at 280 nm. The results were expressed as mg per gram extract (mg g^−1^ extract).

The contents of some phenolic acids and flavonoids in PRHE were determined using a C18 column (4.6 × 250 mm, 5 µm) and eluted with a gradient of 3% acetic acid in water and methanol at a flow rate of 1 mL/min. The phenolic acids and flavonoids were monitored at wavelengths 260, 280, 320 and 360 nm. The amounts of gallic acid, protocatechuic acid, catechin, vanillic acid, epicatechin, *p*-coumaric acid, ferulic acid, rutin and quercetin were calculated by using a calibration curve. The anthocyanins and anthocyanidins contents were determined on the same column with gradient elution of acetonitrile, 4% phosphoric acid in water and methanol and detected at a wavelength of 520 nm. Delphinidin-3-glucoside, cyanidin-3-glucoside, peonidin-3-gluocoside, malvidin-3-glucoside, delphinidin, cyanidin, pelargonidin, peonidin and malvidin were used as the standards. The results were expressed as mg per gram extract (mg g^−1^ extract).

### 4.4. Animals

Male Wistar rats were purchased from the National Laboratory Animal Center, Mahidol University, Salaya, Nakorn-Prathom, Thailand. They were kept in the Animal House, Faculty of Medicine, Chiang Mai University, Chiang Mai, Thailand, under constant conditions (12 h light/dark cycle, 50–60% humidity, at 21–25 °C) and fed diet and tap water ad libitum. They were treated according to experimental protocols approved by The Animal Ethics Committee of Faculty of Medicine, Chiang Mai University (Protocol No. 10/2556 and 14/2558).

### 4.5. Acute Toxicity Test

The acute toxicity test for PRHE was performed according to Organisation for Economic Co-operation and Development (OECD) guideline 425 [35]. Female Wistar rats were randomly divided into 2 groups. The control group was fed with distilled water, while the treated group was given a single dose of 2000 mg kg^−1^ BW of the extract. Body weight, signs of toxicity, behavior and mortality were observed for 24 h after administration. On day 15 of the experiment, all rats were euthanized with 4% isoflurane mixed with oxygen inhalation, for 5 min, in a closed system, at room temperature. The internal organs were excised for weighing and gross pathological observation.

### 4.6. Short-Term Carcinogenicity Test

**Figure 4:**
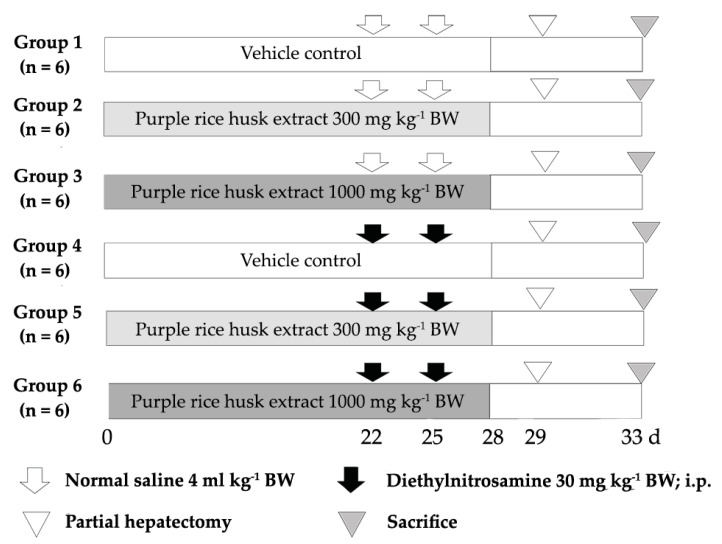
The protocol for the study of the chemopreventive effect of PRHE on the DEN-induced initiation stage of carcinogenesis in rats.

The clastogenicity and anticlastogenicity of the PRHE were determined by using a rat liver micronucleus assay. Male Wistar rats, 4 weeks old (110–120 g), were divided into 6 groups, with 6 rats per group. Group 1 was a negative control, while rats in groups 2 and 3 were orally fed with 300 or 1000 mg kg^−1^ BW of the extract, respectively, for clastogenic determination. To induce hepatic micronucleus formation, rats in groups 4–6 were intraperitoneally (i.p.) injected with 30 mg kg^−1^ BW of DEN. Group 4 was a positive control, whereas groups 5 and 6 were orally treated with 300 or 1000 mg kg^−1^ BW, respectively, for the anticlastogenic study. Rats were treated with the extract for 28 days. Partial hepatectomy (PH) was performed to stimulate hepatocytes into mitosis, on day 29 of the experiment. After PH for 4 days, hepatocytes were isolated by the two-step collagenase perfusion method (Figure 4). The hepatocyte suspensions were stained with 4’,6-diamidino-2-phenylindole (DAPI) solution, and then the number of micronucleated hepatocytes was determined under a fluorescent microscope.

### 4.7. Determination of Phases I and II Xenobiotic-Metabolizing Enzymes

Liver microsomal and cytosolic (100,000 g supernatant) fractions were prepared by using differential centrifugation, and the protein concentrations were determined by the Lowry method.

The cytochrome P450 reductase (CPR) activity in the microsomal fraction was investigated according to the rate of cytochrome c reduction in a reaction mixture containing 5 mM NADPH, 0.3 M potassium phosphate buffer (pH 7.5), 1 mM cytochrome *c* and 50 mM KCN. The activity was measured at 550 nm and calculated by using a molar extinction coefficient of 21 M^−1^ cm^−1^.

The activity of microsomal HO was measured by using hemin as a substrate. The reaction was carried out in the presence of 0.1 M potassium phosphate buffer with 2 mM MgCl_2_ (pH 7.4), 50 mM hemin, 0.8 mM NADPH, 2 mM G-6-P, 0.2 U G-6-PD and biliverdin reductase. The supernatant was determined at 460 and 530 nm. The HO activity was calculated by using an extinction coefficient of 40 mM^−1^ cm^−1^.

The activity of cytosolic NADPH-quinone reductase (NQO) was determined by using 2,6-dichlorophenol-indophenol (DCPIP) as an electron acceptor and by measuring the rate of DCPIP reduction in a reaction mixture containing 0.025 M Tris-HCl buffer (pH 7.4), 1.0 mg/mL BSA, 1% Tween-20, 150 µM FAD, 30 mM NADPH and 24 mM DCPIP. The absorbance of DCPIP was measured at 600 nm. The NQO activity was calculated by using an extinction coefficient of 2.1 × 10^4^ M^−1^ cm^−1^.

The UDP-glucuronyltransferase (UGT) activity was determined by the reaction of a mixture containing 0.1 M Tris buffer, 4 mM MgCl_2_, 20 mM UDP-glucuronic acid and 0.5 mM *p*-nitrophenol (PNP), in a microsomal fraction, at 37 °C, for 20 min. After stopping the reaction with ice-cold 10% TCA, the reaction mixture was centrifuged at 10,000× *g* for 5 min and alkalinized with 0.5 M NaOH. The absorbance was measured at 405 nm. The activity of UGT was calculated by using an extinction coefficient of 18 mM^−1^ cm^−1^.

The activity of cytosolic GST was examined by using 1-chloro-2,4-dinitrobenzoic acid (CDNB) as a substrate. The cytosolic fraction was preincubated with a reaction mixture consisting of 0.2 M phosphate buffer, 10 mM GSH, 10 mM CDNB and a cytosolic fraction. Then, the absorbance at 340 nm was determined and calculated by using a molar extinction coefficient of 9.6 mM^−1^ cm^−1^.

The expression of cytochrome P450 2E1 (CYP2E1; Santa Cruz Biotechnology, Heidelberg, Germany) and glutathione *S*-transferase alpha form (GST-A; Abcam, Cambridge, UK) was determined by Western blot analysis. Protein detection was performed by using horseradish peroxidase conjugated secondary antibodies and a Pierce-enhanced chemiluminescent kit. The intensity of each band was normalized to β-actin and was evaluated with the Image J program.

### 4.8. Medium-Term Liver and Colon Carcinogenicity Test

**Figure 5:**
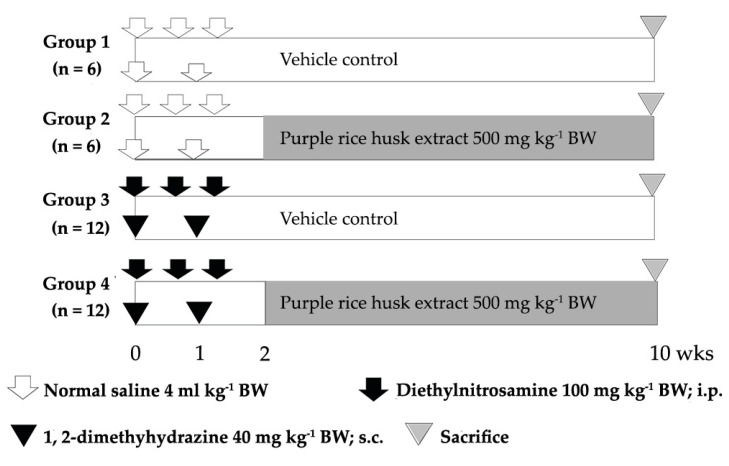
The protocol for the study of the chemopreventive effect of purple rice husk extract on the DEN- and DMH-induced promotion stage of carcinogenesis in rats.

The carcinogenic and anticarcinogenic effects of PRHE were evaluated by using a dual-organ carcinogenicity model according to Punvittayagul et al., 2019 [36]. The treatment protocol is shown in Figure 5. Male Wistar rats, 4 weeks old (110–120 g), were divided into 4 experimental groups. Groups 1 and 2 were injected with 0.9% normal saline solution, while groups 3 and 4 were intraperitoneally injected with 100 mg kg^−1^ BW of diethylnitrosamine (DEN) on day 0, 4 and 9, to induce hepatic preneoplastic lesions and subcutaneous injection of 40 mg kg^−1^ BW of 1,2-dimethylhydrazine (DMH) on day 0 and day 7, to initiate colonic preneoplastic lesions. After DEN and DMH injections for 5 days, groups 2 and 4 were fed with the extract at a dose of 500 mg kg^−1^ BW for 8 weeks, to evaluate carcinogenic and anticarcinogenic effects of the extract. All rats were sacrificed at week 10, using an isoflurane anesthetic, and then the numbers of hepatic GST-P-positive foci and colonic ACF were determined under a light microscope.

### 4.9. Determination of Preneoplastic Lesions in Liver and Colon

The hepatic GST-P-positive foci were determined by an immunohistochemical staining method [37]. Liver sections were deparaffinized and rehydrated. After soaking in 3% H_2_O_2_ and skimmed milk, the slides were incubated with polyclonal anti-rabbit GST-P antibody (MBL, Nagoya, Japan) and with secondary antibody (goat anti-rabbit IgG) conjugated with avidin–biotin peroxidase complex. The brown color was developed with 3,3′-diaminobenzidine (DAB) and then counterstained with hematoxylin. The number of GST-P positive foci larger than 0.2 mm in diameter was measured by using the LAS Interactive Measurement program.

The detection and quantitation of colonic ACF were performed by using methylene blue staining. Briefly, the formalin-fixed colons were cut open along the longitudinal median axis and then stained with 0.2% methylene blue. The number of colonic ACF in each rat was determined under a light microscope.

### 4.10. Determination of Proliferating Cell Nuclear Antigen and Apoptotic Hepatocytes by Double-Staining Immunohistochemistry

The double-staining procedures were performed by using an EnVision Doublestain system from Dako (Glostrup, Denmark). Liver sections were performed for immunohistochemical staining with anti-PCNA antibody (Biolegend, San Diego, CA, USA) and anti-rat GST-P antibody according to manufacturer’s instructions. The number of PCNA-positive cells was counted both inside and in the area surrounding GST-P positive foci. The PCNA labeling index (%) was determined by counting at least 2000 hepatocytes.

Apoptotic hepatocytes were determined by terminal deoxynucleotidyl transferase dUTP nick end labeling (TUNEL) assay. The double immunostaining for TUNEL and GST-P was performed by using an ApopTag Peroxidase in situ kit and an EnVision Doublestain system according to Thumvijit et al. (2014) [34]. The number of apoptotic cells was counted both inside and surrounding the areas of GST-P-positive foci. The apoptotic labeling index (%) was determined by counting at least 2000 hepatocytes.

### 4.11. Statistical Analysis

Data are expressed as mean ± SD of each variable for each group. The statistical significance of the difference between groups was analyzed by one-way analysis of variance with least significant difference for post hoc tests.

## 5. Conclusions

The PRHE presented non-adverse effects in rats, including systemic toxicity, mutagenicity and carcinogenicity. PRHE inhibited the formation of liver micronucleus and hepatic GST-P-positive foci in chemically induced rat hepatocarcinogenesis. These results might indicate that PRHE acted as both a blocking and a suppressing agent. Anthocyanins and vanillic acid might be possible anticarcinogenic compounds in purple rice husk. However, their anticancer activity should be further confirmed. This study contributes to increase the clarification of anticarcinogenic potential of purple rice husk in chemically induced rat liver and colon carcinogenesis. The current data may provide useful information of a waste by-product from purple rice, for utilization in alternative medicine.

## Figures and Tables

**Table 1:** Chemical constituents of purple rice husk extract (PRHE).

Analytes	Content (mg g^−1^ Extract)
α-Tocopherol	0.75 ± 0.02
β-Tocopherol	0.08 ± 0.00
γ-Tocopherol	0.13 ± 0.00
δ-Tocopherol	0.03 ± 0.01
γ-Tocotrienol	0.10 ± 0.00
Protocatechuic acid	0.45 ± 0.01
Vanillic acid	1.53 ± 0.02
*p*-Coumaric acid	0.54 ± 0.00
Ferulic acid	0.06 ± 0.00
Rutin	0.17 ± 0.05
Cyanidin-3-glucoside	0.32 ± 0.00
Peonidin-3-glucoside	0.55 ± 0.01
Malvidin-3-glucoside	0.71 ± 0.02
Delphinidin-3-glucoside	0.04 ± 0.00
Cyanidin	0.56 ± 0.05
Peonidin	0.04 ± 0.00

Value are expressed as mean ± SD.

**Table 2:** The number of hepatic GST-P-positive foci and colonic ACF of rats initiated with diethylnitrosamine and 1,2-dimethylhydrazine.

Treatment	Preneoplastic Lesions			
	Liver		Colon	
	No. of GST-P^+^ foci/cm^2^	Area of GST-P^+^ Foci (mm^2^/cm^2^)	Aberrant Crypt/Rat	Aberrant Crypt/Focus
NSS	0.00 ± 0.00	0.00 ± 0.00	0.00 ± 0.00	0.00 ± 0.00
NSS + PRHE 500 mg kg^−1^ BW	0.00 ± 0.00	0.00 ± 0.00	0.00 ± 0.00	0.00 ± 0.00
DEN + DMH	17.45 ± 3.97 *	1.84 ± 0.74 *	169.56 ± 70.97 *	4.45 ± 1.00 *
DEN + DMH + PRHE 500 mg kg^−1^ BW	11.06 ± 4.89 **	1.37 ± 0.81	226.43 ± 112.61	4.68 ± 0.55

Values are expressed as mean ± SD. DEN: diethylnitrosamine 100 mg kg^−1^ BW, i.p. DMH: 1,2-dimethylhydrazine 40 mg kg^−1^ BW, s.c. NSS: normal saline solution 4 mL kg^−1^ BW. PRHE: purple rice husk extract. * Significantly different from negative control group, *p* < 0.05. ** Significantly different from positive control group, *p* < 0.05.

**Table 3:** Proliferating cell nuclear antigen (PCNA) and apoptotic labeling indices in GST-P-positive foci of rats.

Treatment	PCNA Labeling Index (%)		Apoptotic Labeling Index (%)	
	GST-P-Positive Foci	Surrounding Area	GST-P-Positive Foci	Surrounding Area
NSS	0.00 ± 0.00	2.81 ± 0.52	0.00 ± 0.00	0.30 ± 0.19
NSS + PRHE 500 mg kg^−1^ BW	0.00 ± 0.00	2.07 ± 0.55	0.00 ± 0.00	0.34 ± 0.13
DEN + DMH	6.67 ± 2.81 *	4.20 ± 1.39	0.42 ± 0.37	0.40 ± 0.15
DEN + DMH + PRHE 500 mg kg^−1^ BW	3.38 ± 1.71 **	2.50 ± 0.90 **	0.72 ± 0.44	0.99 ± 0.58 **

Values are expressed as mean ± SD. * Significantly different from negative control group, *p* < 0.05. ** Significantly different from positive control group, *p* < 0.05. DEN: diethylnitrosamine 100 mg kg^−1^ BW, i.p. DMH: 1,2-dimethylhydrazine 40 mg kg^−1^ BW, s.c. NSS: normal saline solution 4 mL kg^−1^ BW. PRHE: purple rice husk extract.

## Data Availability

The data presented in this study are available on request from the corresponding author.

## References

[1] Pourhoseingholi M.A., Vahedi M., Baghestani A.R. (2015). Burden of gastrointestinal cancer in Asia: An overview. Gastroenterol. Hepatol. Bed Bench.

[2] Pandey K.B., Rizvi S.I. (2009). Plant polyphenols as dietary antioxidants in human health and disease. Oxid. Med. Cell. Longev..

[3] Varzakas T., Zakynthinos G., Verpoort F. (2016). Plant food residues as a source of nutraceuticals and functional foods. Foods.

[4] Wang Y., Zhang H., Liang H., Yuan Q. (2013). Purification, antioxidant activity and protein-precipitating capacity of punicalin from pomegranate husk. Food Chem..

[5] Xiang L., Wang Y., Yi X., Wang X., He X. (2016). Chemical constituent and antioxidant activity of the husk of Chinese hickory. J. Funct. Foods.

[6] Barreca D., Laganà G., Leuzzi U., Smeriglio A., Trombetta D., Bellocco E. (2016). Evaluation of the nutraceutical, antioxidant and cytoprotective properties of ripe pistachio (*Pistacia vera* L., variety Bronte) hulls. Food Chem..

[7] Park J.Y., Shin M.S., Kim S.N., Kim H.Y., Kim K.H., Shin K.S., Kang K.S. (2016). Polysaccharides from Korean Citrus hallabong peels inhibit angiogenesis and breast cancer cell migration. Int. J. Biol. Macromol..

[8] Esa N.M., Ling T.B., Peng L.S. (2013). By-products of rice processing: An overview of health benefits and applications. Rice Res..

[9] Wanyo P., Meeso N., Siriamornpun S. (2014). Effects of different treatments on the antioxidant properties and phenolic compounds of rice bran and rice husk. Food Chem..

[10] Nilnumkhum A., Punvittayagul C., Chariyakornkul A., Wongpoomchai R. (2017). Effects of hydrophilic compounds in purple rice husk on AFB_1_-induced mutagenesis. Mol. Cell Toxicol..

[11] Punvittayagul C., Sringarm K., Chaiyasut C., Wongpoomchai R. (2014). Mutagenicity and antimutagenicity of hydrophilic and lipophilic extracts of Thai northern purple rice. Asian Pac. J. Cancer Prev..

[12] Busat S., Siriamornpun S. (2020). Phenolic acids and antioxidant activities in husk of different Thai rice varieties. Food Sci. Technol. Int..

[13] Chariyakornkul A., Punvittayagul C., Taya S., Wongpoomchai R. (2019). Inhibitory effect of purple rice husk extract on AFB_1_-induced micronucleus formation in rat liver through modulation of xenobiotic metabolizing enzymes. BMC Complement. Altern. Med..

[14] Chung N.J., Choi K.C., Lee S.A., Baek J.A., Lee J.C. (2015). Rice hull extracts inhibit proliferation of MCF-7 cells with G1 cell cycle arrest in parallel with their antioxidant activity. J. Med. Food.

[15] Kim S.J., Park H.R., Park E., Lee S.C. (2007). Cytotoxic and antitumor activity of momilactone B from rice hulls. J. Agric. Food Chem..

[16] Sankam P., Punvittayagul C., Sringam K., Chaiyasut C., Wongpoomchai R. (2013). Antimutagenicity and anticlastogenicity of glutinous purple rice hull using in vitro and in vivo testing systems. Mol. Cell Toxicol..

[17] Xu Z., Hua N., Godber J.S. (2001). Antioxidant activity of tocopherols, tocotrienols, and gamma-oryzanol components from rice bran against cholesterol oxidation accelerated by 2,2′-azobis(2-methylpropionamidine) dihydrochloride. J. Agric. Food Chem..

[18] Tan B.L., Norhaizan M.E. (2017). Scientific evidence of rice by-products for cancer prevention: Chemopreventive properties of waste products from rice milling on carcinogenesis in vitro and in vivo. Biomed Res. Int..

[19] Chen P.N., Chu S.C., Chiou H.L., Chiang C.L., Yang S.F., Hsieh Y.S. (2005). Cyanidin-3-glucoside and peonidin-3-glucoside inhibit tumor cell growth and induce apoptosis in vitro and suppress tumor growth in vivo. Nutr. Cancer.

[20] Tomankova E., Balik J., Soural I., Bednar P., Papouskova B. (2016). Colour and antioxidant properties of malvidin-3-glucoside and vitisin A. Acta Aliment..

[21] Khoo H.E., Azlan A., Tang S.T., Lim S.M. (2017). Anthocyanidins and anthocyanins: Colored pigments as food, pharmaceutical ingredients, and the potential health benefits. Food Nutr. Res..

[22] Steward W.P., Brown K. (2013). Cancer chemoprevention: A rapidly evolving field. Br. J. Cancer.

[23] Kang J.S., Wanibuchi H., Morimura K., Gonzalez F.J., Fukushima S. (2007). Role of CYP2E1 in diethylnitrosamine-induced hepatocarcinogenesis in vivo. Cancer Res..

[24] Park Y.C., Lee S., Cho M.H. (2014). The simplest flowchart stating the mechanisms for organic xenobiotics-induced toxicity: Can it possibly be accepted as a “central dogma” for toxic mechanisms?. Toxicol. Res..

[25] Mason E.F., Rathmell J.C. (2011). Cell metabolism: An essential link between cell growth and apoptosis. Biochim. Biophys. Acta.

[26] Al-Ishaq R.K., Overy A.J., Büsselberg D. (2020). Phytochemicals and gastrointestinal cancer: Cellular mechanisms and effects to change cancer progression. Biomolecules.

[27] Hollman P.C.H. (2004). Absorption, bioavailability, and metabolism of flavonoids. Pharm. Biol..

[28] Dai J., Mumper R.J. (2010). Plant phenolics: Extraction, analysis and their antioxidant and anticancer properties. Molecules.

[29] Li Y.W., Wang D., Li X.G., Jin Y. (2014). Anthocyanins extracted from Chinese blueberry and its anticancer effects on HepG2 cells. Adv. Mater. Res..

[30] Tanaka T., Tanaka T., Tanaka M. (2011). Potential cancer chemopreventive activity of protocatechuic acid. J. Exp. Clin. Med..

[31] Krajka-Kuzniak V., Kaczmarek J., Baer-Dubowska W. (2008). Effect of naturally occurring phenolic acids on the expression of glutathione *S*-transferase isozymes in the rat. Food Chem. Toxicol..

[32] Gong J., Zhou S., Yang S. (2019). Vanillic acid suppresses HIF-1α expression via inhibition of mTOR/p70S6K/4E-BP1 and Raf/MEK/ERK pathways in human colon cancer HCT116 cells. Int. J. Mol. Sci..

[33] Yang C.S., Luo P., Zeng Z., Wang H., Malafa M., Suh N. (2020). Vitamin E and cancer prevention: Studies with different forms of tocopherols and tocotrienols. Mol. Carcinsog..

[34] Jia Z., Tang M., Wu J. (1999). The determination of flavonoid contents in mulberry and their scavenging effects on superoxide radicals. Food Chem..

[35] OECD (2008). Test No: 425 Acute Oral Toxicity: Up and Down Procedure. OECD Guidelines for the Testing of Chemicals, Section 4.

[36] Punvittayagul C., Chariyakornkul A., Chewonarin T., Jarukamjorn K., Wongpoomchai R. (2019). Augmentation of diethylnitrosamine-induced early stages of rat hepatocarcinogenesis b 1,2-dimethylhydrazine. Drug Chem. Toxicol..

[37] Thumvijit T., Taya S., Punvittayagul C., Peerapornpisal Y., Wongpoomchai R. (2014). Cancer chemopreventive effect of *Spirogyra neglecta* (Hassall) Kützing on diethylnitrosamine-induced hepatocarcinogenesis in rats. Asian Pac. J. Cancer Prev..

